# Reagent controlled addition of chiral sulfur ylides to chiral aldehydes

**DOI:** 10.1186/1860-5397-1-4

**Published:** 2005-08-26

**Authors:** Varinder K Aggarwal, Jie Bi

**Affiliations:** 1 School of Chemistry, University of Bristol, Cantock's Close, Bristol BS8 1TS, UK

## Abstract

The degree of reagent and substrate control in the reaction of chiral sulfur ylides with chiral aldehydes has been investigated. Specifically, the reactions of the two enantiomers of the chiral benzyl sulfonium salt **1** with glyceraldehyde acetonide were studied in detail. Of the two new stereogenic centers created, it was found that the C^1^ stereochemistry was largely controlled by the reagent, whereas control at the C^2^ center was dependent on the aldehyde used. In one case, the *trans* isomer was produced *via* reversible formation of the intermediate betaine, whereas in the alternative case, the C^2^ center was under Felkin Anh/Cornforth control through non-reversible formation of the betaine. Thus, the aldehyde stereocenter influenced the degree of reversibility in betaine formation, which impacted on the stereocontrol at the C^2^ position.

## Introduction

**Scheme 1:**
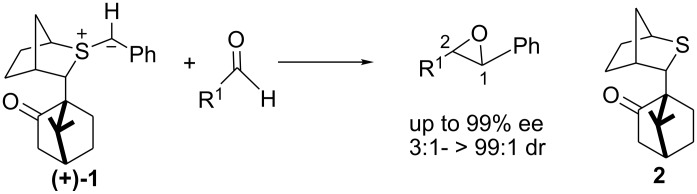


The reaction of chiral sulfur ylides with carbonyl compounds, operating in either a catalytic or stoichiometric mode, have emerged as a useful and powerful method in the arsenal of asymmetric transformations.[1] Indeed, near perfect levels of asymmetric induction with high diastereocontrol have been achieved with aromatic aldehydes (Scheme 1). In such reactions, the C^1^ stereochemistry is controlled by ylide conformation, face selectivity, and the degree of reversibility in formation of the *anti* betaine (high reversibility results in low stereocontrol) whilst the C^2^ stereochemistry is controlled by the degree of reversibility in *syn* betaine formation (high reversibility leads to high diastereocontrol in favor of the *trans* epoxide).[2]

**Scheme 2:**
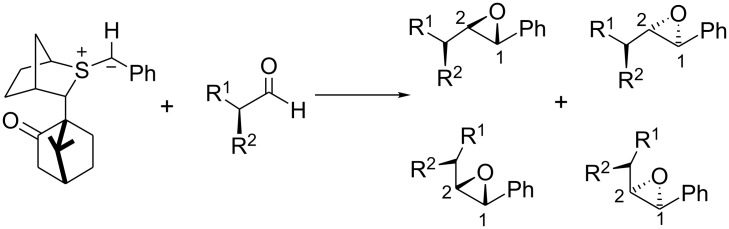


We questioned whether the high level of reagent control clearly shown by sulfide **2** could be exploited in reactions with chiral aldehydes and to what extent it might dominate over substrate control (Scheme 2).[3] Again C^1^ stereochemistry should be controlled by ylide conformation, face selectivity, degree of reversibility in *anti* betaine formation, and is not expected to be significantly influenced by the nature (or stereochemistry) of the aldehyde. Therefore, very high levels of reagent control can be expected for the C^1^ stereogenic centre. As before, C^2^ stereochemistry is controlled by the degree of reversibility in *syn* betaine formation, and if the addition is non-reversible, will be influenced by the Felkin-Anh selectivity. The degree of reversibility in betaine formation is affected by the nature (and perhaps stereochemistry) of the aldehyde employed.

**Scheme 3:**
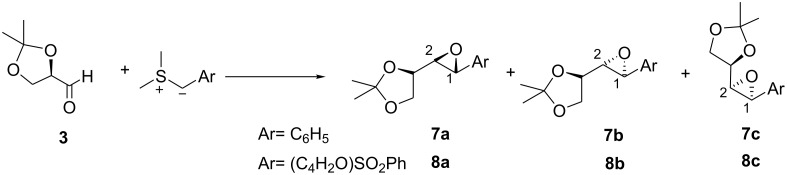


**Scheme 4:**
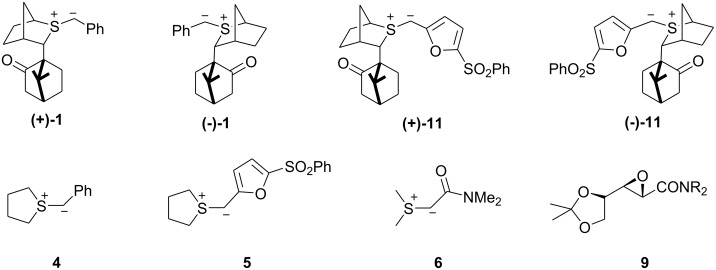


We therefore examined the reactions of chiral sulfur ylides with chiral aldehydes in order to establish the extent of reagent and substrate control in such systems and chose glyceraldehyde **3** as the substrate (Scheme 3, Scheme 4).

## Results and Discussion

**Scheme 5:**
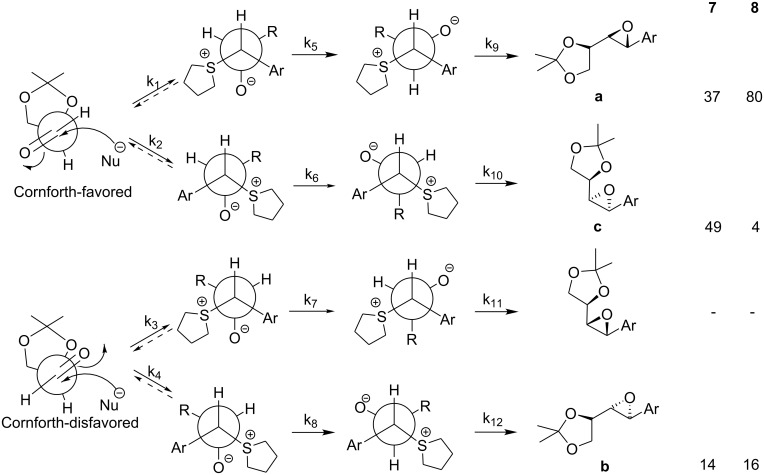


The reaction of glyceraldehyde acetonide **3** with the achiral sulfonium salt in the presence of the P_2_ base (P_2_ = *N,N,N',N'*-tetramethyl-*N"*-[tris(dimethylamino)-phosphoranylidene]-phosphoric triamide ethylimine,) [4–5] (sulfur ylide **4**) was initially investigated to establish the degree of substrate control. This furnished a mixture of 3 epoxides **7a**, **7b**, and **7c** in a 37:14:49 ratio (Table 1, entry 1). The *cis* and *trans* isomers are easily distinguished by the vicinal coupling constants (*cis* >*trans*). Assignment of the two trans diastereomers has been based on (i) the model for addition of the two enantiomers of the chiral sulfide to aldehydes and (ii) comparison of the H^2^-H^3^ coupling constants found for the major and minor diastereomers of the glycidic amide **9** [*J* (major isomer) 5.4 Hz, *J* (minor isomer) 3.5 Hz] (see ref 7) with **7a** (*J* 5.8 Hz) and **7b** (*J* 4.4 Hz). Comparison of the 13C NMR of the methyl groups of the acetonide of **9** with **7a/b** and **8a/b** also provide a consistent picture for structural assignment. The large amount of *cis* epoxide obtained indicated that the reaction was largely non-reversible and under such conditions, the degree of substrate control could be established. Thus, the degree of substrate control, [6] which is given by the ratio of the 2*S*:2*R* isomers, was 86:14 (**7a**+**7c**: **7b**). Interestingly, using the more stable ylide **5**, which now reacted much more reversibly as evidenced by the higher *trans* selectivity (96: 4), the C^2^ control was similar (84:16) (**8a**+**8c**: **8b**) to the reaction under kinetic control (Table 1, entry 2). This indicated that the rate of bond rotation or the rate of the ring closure of the two *anti* betaines (k_5_ and k_7_ or k_9_ and k_11_) was very similar and that the ratio of epoxides obtained was therefore governed by the rates of betaine formation (k_1_ and k_3_) which again is determined by the degree of Felkin-Anh/Cornforth control by the substrate (Scheme 5). These experiments showed that in the absence of reagent control, low selectivity was observed with semi-stabilized ylides reacting non-reversibly but good levels of diastereocontrol could be achieved with more stabilized ylides reacting reversibly. Interestingly, the amide-stabilized ylide **6**, for which betaine formation is expected to be even more reversible, has been reported to give exclusively the *trans* epoxide **9** but again as an 86: 14 ratio of diastereoisomers.[7]

**Table 1:** Epoxidation of (+)-Glyceraldehyde with Sulfur Ylides

Entry	Sulfur ylide	other conditions^a^	Yield^b^	diastereoselectivity *d.r.*^c^ (a:b:c)

1	**4**	---	72 %	37:14:49
2	**5**	---	62 %	80:16:4
3	(+)-**1**	---	63 %	89:5:6
4	(+)-**1**	LiCl	64 %	96:0:4
5	(-)-**1**	---	56 %	1:21:78
6	(-)-**1**	LiCl	61 %	2:32:66
7	(+)-**11**	---	49 %	53:33:14
8	(-)-**11**	---	52 %	52:42:6

a: Reactions were carried out in CH_2_Cl_2_ at -78°C, using EtP_2_ Base to form the sulfur ylide; b: Isolated yields; c: Diastereomer ratios were determined by 1H NMR.

We then turned our attention to reactions of the two chiral sulfur ylide enantiomers ((+)-**1**/(-)-**1**) with aldehyde **3** (Table 1, entry 3 and 5). Interestingly, in both cases, high levels of selectivity for the C_1_ position were observed (C_1_(*R*):C_1_(*S*) (**7a**: **7b**+**7c**), 89:11 for (+)-**1**; 1:99 for (-)-**1**), indicating that high degrees of reagent control were operative in such systems.

**Scheme 6:**
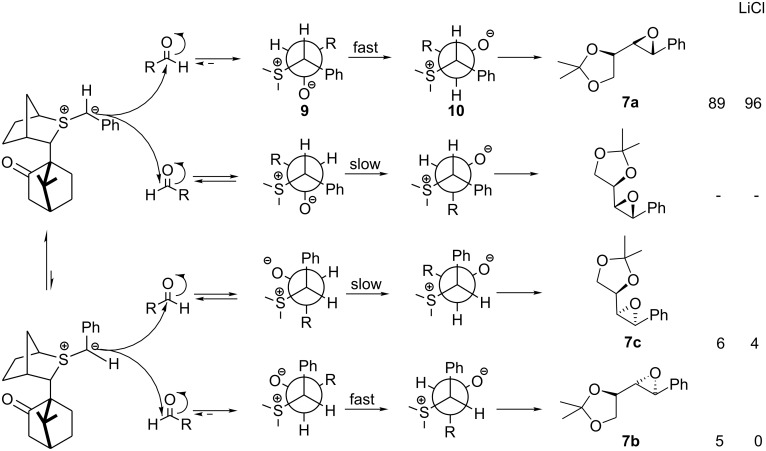


The high degree of *trans* selectivity observed with (+)-**1** (94:6) is indicative of a high degree of reversibility in *syn* betaine formation (Scheme 6). We suspected that the lower C^1^ selectivity observed for (+)-**1** relative to (-)-**1** resulted from partial reversibility in *anti* betaine formation. In fact when both *syn* and *anti* betaine formation is highly reversible, whilst high *trans* selectivity is observed, selectivity at C^1^ is eroded. This is illustrated with the even more stabilized chiral ylide **11** which, because of its stability, reacted even more reversibly with glyceraldehydes **3** giving even lower C^1^ selectivity: 53: 47 for the reaction of (+)-**11** and 52: 48 for the reaction of (-)-**11** (Table 1, entry 8, entry 9). From earlier studies we had established that the degree of reversibility in betaine formation could be influenced by the degree of solvation of the intermediate alkoxide. The initially formed betaine **9** with charges gauche to each other can either undergo bond rotation to betaine **10** followed by ring closure to give the epoxide **7a** or reversion to the ylide and the aldehyde. Solvation of the alkoxide **9** reduces the barrier to bond rotation rendering reactions less reversible. We therefore examined the same reactions in the presence of LiCl (Table 1, entries 4, 6). Now increased C^1^ selectivity (C_1_(*R*):C_1_(*S*), 96:4) was observed with the reaction of (+)-**1**, furnishing essentially a single diastereomer in good yield.

**Scheme 7:**
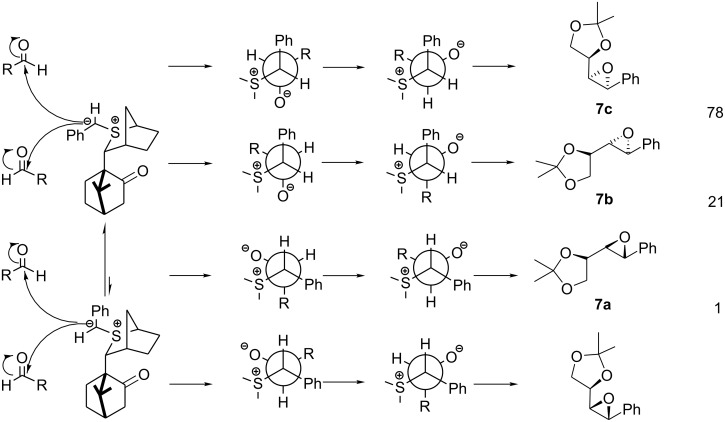


In contrast to the reaction of (+)-**1**, which reacted partially-reversibly with aldehyde **3**, (-)-**1** reacted essentially non-reversibly as evidenced by the unusually high degree of *cis* selectivity (the trans isomer is normally favoured in these types of reactions) observed (78:22). In fact the *cis* isomer **7c** was the major diastereomer formed (Scheme 7). Under such non-reversible conditions, the C^1^ stereochemistry was controlled by the reagent (C_1_(*R*):C_1_(*S*), 1:99) and the C^2^ stereochemistry was controlled by the substrate (C_2_(*S*):C_2_(*R*), 79:21). Interestingly, the observed C^2^ selectivity was similar to that obtained with achiral sulfur ylides, and the observed C^1^ selectivity was similar to that obtained with achiral aldehydes. Thus, when the reaction is non-reversible, the C^1^ stereochemistry, which is controlled by the reagent, does not influence the C^2^ stereochemistry. As such the selectivity at C^2^ is controlled in the betaine formation step which is essentially completely controlled by the substrate and not the ylide. In other words the C^1^ and C^2^ selectivities are controlled essentially independently when betaine formation is non-reversible. Although we are able to modify the reaction conditions to make betaine formation less reversible, no methods are available at present to make it more reversible.

In conclusion, reaction of the chiral sulfur ylide **1** with the chiral aldehyde glyceraldehyde acetonide **3** gives high reagent control at the C^1^ centre. Control at the C^2^ centre is more finely balanced and is dependent on whether betaine formation is reversible or not. If betaine formation is reversible, the stereochemistry at C^2^ is controlled by the ylide, delivering the *trans* diastereomer. If betaine formation is non-reversible, the stereochemistry is influenced by pre-existing stereochemistry of the chiral aldehyde (Felkin-Anh/Cornforth control). In this case the C^1^ and C^2^ stereochemistries are independently controlled with little impact on each other. The factors that we previously noted that influenced reversibility in betaine formation included ylide stability, solvation of the metal alkoxide and steric hindrance around the aldehyde and ylide.[2] We now add another factor to this growing list: stereochemistry of the aldehyde. Evidently, in one isomer, the groups between the aldehyde and ylide mesh well together allowing facile bond rotation from the initially formed gauche betaine to the *trans* betaine rendering the reaction non-reversible. In the other isomer the groups clash resulting in a higher barrier to bond rotation thus leading to reversible betaine formation. In many respects it is surprising that the subtle effect of stereochemistry of the aldehyde has such a significant impact on the bond rotation step and therefore reversibility of betaine formation.

## Supporting Information

File 1 Experimental details
