# Supplementary material for: Reagent controlled addition of chiral sulfur ylides to chiral aldehydes
Source: Beilstein J Org Chem. 2005 Aug 26;1:4. doi: 10.1186/1860-5397-1-4 (PMC1399452; doi:10.1186/1860-5397-1-4)
Supplement: File 1 — Experimental details [file Beilstein_J_Org_Chem-01-04-s001.doc]

**Experimental**

**General Procedure for Epoxidation at Low Temperature using P_2_ Base**

To a solution of the sulfonium salt (0.50 mmol) in anhydrous dichloromethane (0.7 mL) was added *N*,*N*,*N′*,*N′*-tetramethyl-*N″*-[tris(dimethylamino)phosphoralidene] phosphoric triamide ethylimine (0.50 mmol) at -78 °C. After stirring for 10–15 min a solution of the desired aldehyde (0.50 mmol) in anhydrous dichloromethane (1.0 mL) was added dropwise and the reaction mixture was stirred for 1.5 hours at -78 °C. After addition of water (20 mL), the organic phase was separated and the aqueous phase extracted with CH_2_Cl_2_ (2 × 20 mL). The organic phases were combined, dried over magnesium sulfate, filtered and concentrated in vacuum. Purification by column chromatography yields the pure epoxides as one *cis* diastereomer and a mixture of two *trans* diastereomers.

**1-Phenyl-2-[(*R*)-2,2-dimethyl-1,3-dioxolan-4-yl] oxirane (7).^1^**

The mixture of two *trans* epoxides (1*R*,2*S*)-1-Phenyl-2-[(*R*)-2,2-dimethyl-1,3- dioxolan-4-yl] oxirane (**7a**) and (1*S*,2*R*)-1-Phenyl-2-[(*R*)-2,2-dimethyl-1,3-dioxolan -4-yl] oxirane (**7b**) was obtained as a pale yellow oil, R*f*(10 %EtOAc in petroleum) 0.31; υ_max_/cm^-1^ 2987 (CH), 1253, 1212, 840; δ_H_ (400 MHz, CDCl_3_) **7a**: 1.40 (3H, s, CH_3_), 1.46 (3H, s, CH_3_), 3.09-3.11 (1H, dd, *J* 5.8 Hz and 2.0 Hz, C**^2^**H), 3.80 (1H, d, *J* 2.0 Hz, C**^1^**HPh), 3.98-4.01 (1H, dd, *J* 7.8 Hz and 5.8 Hz, C**^4^***H*H), 4.02-4.06 (1H, q, *J* 5.8Hz, C**^3^**H), 4.18 (1H, dd, *J* 7.8 Hz and 5.8 Hz, C**^4^**H*H*), 7.27-7.38 (5H, m, ArH); **7b**: 1.40 (3H, s, CH_3_), 1.46 (3H, s, CH_3_), 3.08-3.10 (1H, dd, *J* 4.4 Hz and 2.0 Hz, C**^2^**H), 3.86 (1H, d, *J* 2.0 Hz, C**^1^**HPh), 3.91-3.95 (1H, dd, *J* 8.3 Hz and 6.4 Hz, C**^4^**H*H*), 4.14-4.17 (1H, dd, *J* 8.3 Hz and 6.4 Hz, C**^4^***H*H), 4.19-4.22 (1H, td, *J* 6.4 Hz, 4.4 Hz, C**^3^**H), 7.27-7.38 (5H, m, ArH); δ_C_ (100 MHz, CDCl_3_) **7a**: 25.2 (CH_3_), 26.5 (CH_3_), 57.3 (CH), 62.2 (CH), 66.8 (CH), 75.9 (CH), 110.0 (C), 125.7 (CH), 128.5 (2CH), 136.4 (C); **7b**: 25.6 (CH_3_), 26.2 (CH_3_), 55.4 (CH), 61.9 (CH), 66.0 (CH), 75.0 (CH), 110.1 (C), 125.7 (CH), 128.5 (2CH), 136.6 (C); *m*/*z* (CI) 221 (MH^+^, 3 %), 163 (34), 121(47), 101 (97), 59 (100).

The only one *cis* epoxide (1*S*,2*S*)-1-Phenyl-2-[(*R*)-2,2-dimethyl-1,3-dioxolan-4-yl] oxirane (**7c**) was obtained as a pale yellow oil, R*f*(10 %EtOAc in petroleum) 0.33; υ_max_/cm^-1^ 2987 (CH), 1253, 1212, 840; α_D_^25^ +34.0 (CHCl_3_); δ_H_ (400 MHz, CDCl_3_) 1.22 (3H, s, CH_3_), 1.45 (3H, s, CH_3_), 3.25-3.28 (1H, dd, *J* 8.3 Hz and 4.2 Hz, C**^2^**H), 3.53-3.58 (1H, ddd, *J* 8.3 Hz, 6.3 Hz and 5.0 Hz, C**^3^**H), 4.01-4.05 (1H, dd, *J* 8.8 Hz and 6.3 Hz, C**^4^***H*H), 4.06-4.10 (1H, dd, *J* 8.8 Hz and 5.0 Hz, C**^4^**H*H*), 4.21 (1H, d, *J* 4.2 Hz, C**^1^**HPh), 7.27-7.38 (5H, m, ArH); δ_C_ (100 MHz, CDCl_3_) 25.2 (CH_3_), 26.8 (CH_3_), 57.4 (CH), 59.0 (CH), 67.7 (CH_2_), 71.6 (CH), 109.8 (C), 126.4 (CH), 127.9 (CH), 128.3 (CH), 134.3 (C); *m*/*z* (CI) 221 (MH^+^, 2 %), 163 (10), 117(17), 101 (100), 59 (26), (Found: M^+^, 220.1099. C_13_H_16_O_3_ requires M^+^, 220.2676).

**1-[5-(Phenylsulfonyl)-2-furyl]-2-[(*R*)-2,2-dimethyl-1,3-dioxolan-4-yl]oxirane (8).**

The mixture of two *trans* epoxides (1*S*,2*R*)-1-[5-(phenylsulfonyl)-2-furyl]-2-[(*R*)-2,2- dimethyl-1,3-dioxolan-4-yl]oxirane (**8a**) and (1*R*,2*S*)-1-[5-(phenylsulfonyl)-2- furyl]-2-[(*R*)-2,2-dimethyl-1,3-dioxolan-4-yl]oxirane (**8b**) was obtained as a pale yellow oil, R*f*(30 %EtOAc in petroleum) 0.33; υ_max_/cm^-1^ 2988 (CH), 1330, 1245, 1142, 809; δ_H_ (400 MHz, CDCl_3_) **8a**: 1.36 (3H, s, CH_3_), 1.44 (3H, s, CH_3_), 3.43-3.45 (1H, dd, *J* 5.6 Hz and 2.0 Hz, C**^2^**H), 3.82 (1H, d, *J* 2.0 Hz, C**^1^**H), 3.94-3.97 (1H, dd, *J* 8.3 Hz and 5.6 Hz, C**^4^**H*H*), 3.99-4.03 (1H, dt, *J* 6.3 Hz, 5.6 Hz, C**^3^**H), 4.14-4.17 (1H, dd, *J* 8.3 Hz and 6.3 Hz, C**^4^***H*H), 6.47-6.49 (1H, m, C**^5^**H), 7.16-7.18 (1H, m, C**^6^**H), 7.53-7.66 (3H, m, ArH**^meta&para^**), 7.97-8.01 (2H, m, ArH**^ortho^**); **8b**: 1.40 (3H, s, CH_3_), 1.46 (3H, s, CH_3_), 3.43-3.45 (1H, dd, *J* 5.6 Hz and 2.0 Hz, C**^2^**H), 3.90 (1H, d, *J* 2.0 Hz, C**^1^**H), 3.94-3.91 (1H, dd, *J* 8.3 Hz and 6.3 Hz, C**^4^**H*H*), 4.13-4.17 (1H, dd, *J* 8.3 Hz and 6.3 Hz, C**^4^***H*H), 4.21-4.25 (1H, td, *J* 6.3 Hz, 5.6 Hz, C**^3^**H), 6.47-6.49 (1H, m, C**^5^**H), 7.16-7.18 (1H, m, C**^6^**H), 7.53-7.66 (3H, m, ArH**^meta&para^**), 7.97-8.01 (2H, m, ArH**^ortho^**); δ_C_ (100 MHz, CDCl_3_) **8a**: 25.2 (CH_3_), 26.5 (CH_3_), 50.2 (CH), 59.9 (CH), 66.7 (CH_2_), 75.0 (CH), 110.3 (C), 110.7 (CH), 118.3 (CH), 128.0 (C), 129.4 (C), 133.9 (CH), 139.9 (CH), 150.1 (CH), 155.4 (CH); **8b**: 25.6 (CH_3_), 26.1 (CH_3_), 48.7 (CH), 59.4 (CH), 66.0 (CH_2_), 73.9 (CH), 110.3 (C), 110.6 (CH), 118.3 (CH), 128.0 (C), 129.4 (C), 133.9 (CH), 139.8 (CH), 150.1 (CH), 155.5s (CH); *m*/*z* (CI) 351 (MH^+^, 3 %), 237 (44), 125 (100), 101 (95).

The only one *cis* epoxide (1*R*,2*R*)-1-[5-(phenylsulfonyl)-2-furyl]-2-[(*R*)-2,2- dimethyl-1,3-dioxolan-4-yl]oxirane (**8c**) was obtained as a pale yellow oil.: R*f*(30 %EtOAc in petroleum) 0.34; υ_max_/cm^-1^ 2988 (CH), 1330, 1245, 1142, 809; α_D_^25^ +37.5 (CHCl_3_); δ_H_ (400 MHz, CDCl_3_) 1.25 (3H, s, CH_3_), 1.43 (3H, s, CH_3_), 3.26-3.29 (1H, dd, *J* 8.1 Hz and 4.0 Hz, C**^2^**H), 3.81-3.86 (1H, ddd, *J* 8.1 Hz, 6.3 Hz and 4.9 Hz, C**^3^**H), 4.01 (1H, d, *J* 4.0 Hz, C**^1^**H), 4.03-4.06 (1H, dd, *J* 8.8 Hz and 4.9 Hz, C**^4^***H*H), 4.10-4.14 (1H, dd, *J* 6.3 Hz and 8.8 Hz, C**^4^**H*H*), 6.47 (1H, m, C**^5^**H), 7.17-7.18 (1H, m, C**^6^**H), 7.53-7.66 (3H, m, ArH**^meta&para^**), 7.99-8.01 (2H, m, ArH**^ortho^**); δ_C_ (100 MHz, CDCl_3_) 25.0 (CH_3_), 26.8 (CH_3_), 50.9 (CH), 59.0 (CH), 67.8 (CH_2_), 72.5 (CH), 110.0 (C), 111.1 (CH), 118.0 (CH), 127.9 (C), 129.3 (C), 133.8 (CH), 139.7 (CH), 150.1 (CH), 154.2 (CH); *m*/*z* (CI) 351 (MH^+^, 6 %), 291 (19), 125 (14), 101 (100), (Found: M^+^, 350.0823. C_17_H_18_SO_6_ requires M^+^, 350.3826).

**Reference:**

(1) This compound was previously prepared as a mixture of three (two *trans* and one *cis*) isomers, but was not fully characterized (only ^1^H NMR of the mixture was given). See: Aggarwal, V. K.; Abdel-Rahman, H.; Li, F.; Jones, R.; Standen, M. *Chem. Europ. J.* **1996**, *2*, 1024-1030.
